# Fire regimes, fire experiments and alternative stable states in mesic savannas

**DOI:** 10.1111/nph.17331

**Published:** 2021-05-02

**Authors:** Elmar M. Veenendaal, Mireia Torello‐Raventos, Heloisa S. Miranda, Naomi M. Sato, Thomas A. J. Janssen, Frank van Langevelde, Jon Lloyd

**Affiliations:** ^1^ Plant Ecology and Nature Conservation Group Wageningen University Wageningen 6700 AA the Netherlands; ^2^ School of Marine and Environmental Sciences James Cook University Cairns Qld 4870 Australia; ^3^ Departmento de Ecologia Universidade de Brasilia Brasilia DF 70910‐900 Brazil; ^4^ Department of Earth Sciences, Cluster Earth and Climate Vrije Universiteit Amsterdam Amsterdam the Netherlands; ^5^ Wildlife Ecology and Conservation Group Wageningen University Wageningen 6700 AA the Netherlands; ^6^ School of Life Sciences University of KwaZulu‐Natal Westville Campus Durban 4000 South Africa

**Keywords:** alternative stable states, feedbacks, fire ecology, forest, savannah

## A response to Laris & Jacobs (2021) ‘On the problem of *natural* savanna fires’

In their comment on Veenendaal *et al*. (2018), Laris & Jacobs (2021; in this issue of *New Phytologist*, pp. 11–13) question the appropriateness of fire experiments to simulate effects of fire on tropical vegetation cover as well as objecting to our use of the word ‘natural’ to describe nonanthropogenic fire regimes. They also challenge some of the conclusions we drew as regards the likelihood of fire‐mediated feedbacks causing alternate stable states (ASS) in forest–savanna transitions.

First we would like to note that, as has also been done by others including Furley *et al*. (2008) in what Laris and Jacobs describe as an ‘excellent paper’, we used the term ‘natural fire regime’ as a contrast to the often very short (annual) fire return times as used in many of the classical fire experiments. This was with a full understanding that quantification of any ‘natural’ fire return time in the field is a necessarily complex problem, depending on factors such as spatial scale of measuring, mode of detection, data analysis technique and land use/type, etc. (Archibald *et al.,* 2010; Alencar *et al.,* 2011; Oliveras *et al.,* 2013; Smit *et al.,* 2013; Giglio & Schroeder, 2014; Beringer *et al.,* 2015; Smit & Prins, 2015). To give a range, generally, for open savannas (grasslands and shrub savannas), fire return times may oscillate between 1 and 5 yr (Oliveras *et al.,* 2013). In savanna woodlands, fire frequencies may vary widely, but are in the range 2–10 yr for Africa (Archibald *et al.,* 2010; Smit & Prins, 2015) and Australia (Russell‐Smith *et al.,* 1998; Spessa *et al.,* 2005) and 6–12 yr in South America (Pereira *et al.,* 2014). Regional variation in fire return intervals tends to be largest for tall savanna woodlands and dry forests, with commonly observed values ranging from *c*. 1–4 yr in West Africa to 10 yr in southern Africa (Furley *et al.,* 2008; Janssen *et al.,* 2018). Tropical forests usually have long fire return intervals, varying from 10 yr to decadal or even centennial/millennial intervals (Hall & Swaine, 1981; Hope & Tulip, 1994; Cochrane *et al.,* 1999; Cochrane, 2003; Archibald *et al.,* 2010). In short: a lot can be said about what constitutes a typical fire regime, but as specifically stated in Veenendaal *et al*. (2018), we used the 4‐yr interval as a demonstration of a reasonable norm for fire return times in the absence of significant human intervention and at a scale where fire–vegetation interaction processes play out. Here we would also like to note that – contrary to the claims of Laris and Jacobs – there was no misquotation here as Furley *et al*. (2008) actually wrote that ‘natural fires typically break out in the late dry season with a mean fire return interval (1941–96) of 4.5 years’.

In terms of the importance of our supposed neglect of anthropic regimes, we also would like to note that we stated in our paper that: ‘Indeed, perhaps we should not think of nonanthropic fire regimes as being in any way representative of the current fire regime of the savanna lands with, in particular in Africa, human influences on fire patterns now being the dominating effect for all but the most strictly protected areas.’ Indeed, lest Laris and Jacobs’s comment leads to any confusion pertaining to this issue, we point out that throughout Veenendaal *et al*. (2018) we on many occasions clearly contrasted the effect of anthropic and nonanthropic fire regimes on tropical vegetation structure. However, that is not what our paper was specifically about.

Of course, we do not dispute that the influence of humankind on savanna fires reaches back possibly as far as 400 000 yr. However, considering population fluctuations over this long period, human influence must have varied considerably with the magnitude of any effect also varying from continent to continent. Further, as we also noted in our paper, the often interacting effects of humans and variation in climate are inevitably difficult to disentangle and with the quantitative analysis of the exact impact of human fires in terms of vegetation structure being complex and therefore understandably lacking in Laris and Jacobs’s comment. Of relevance here and as already mentioned in our paper, many eminent scientists of the first half of the twentieth century (namely Aubreville, Stebbing and Schantz to name but a few; see also Laris & Wardell, 2006) had the impression that large parts of the African landscape would have been otherwise covered by forest were it not for long‐term concerted human action. However, the associated ‘derived savanna concept’ has on many occasions been shown to be blatantly wrong (Fairhead & Leach, 1996). Indeed, any quantitative evidence for fire effects on tropical vegetation cover (on which concepts such as ‘fire derived savanna’ hinge) must necessarily come from manipulated experiments. Aubreville himself well understood this point and, contrary to what is inferred by Laris and Jacobs’s comment, although we did indeed state that most early fire experiments were designed with applied management questions in mind, we never in any way suggested that Aubreville’s original fire experiment was itself such a management‐orientated exercise.

**Fig. 1:**
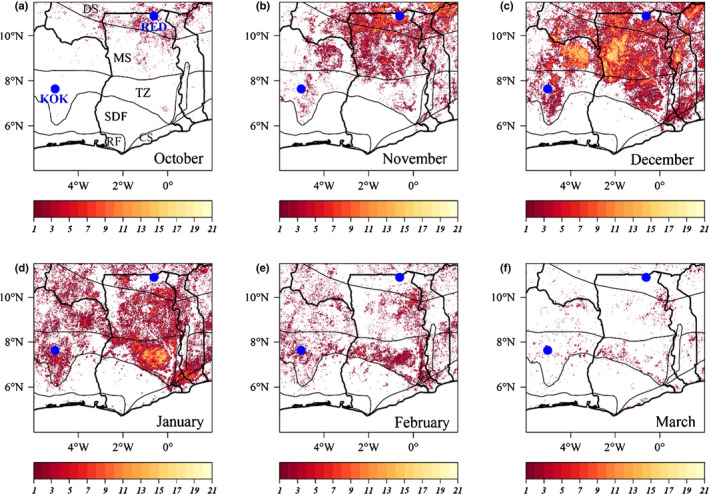
Monthly fire seasonality in the dry season in West Africa in relation to the Red Volta and Kokondekro Fire experiments. The pixel burn count between November 2000 and December 2020 was retrieved from the MODIS burned area monthly product (MCD64A1; Giglio *et al.,* 2018) at 500‐m spatial resolution. The locations of two fire experiments, at Red Volta (RED) in Ghana and Kokondekro (KOK) in Ivory Coast, are indicated in blue. Thick black lines are country borders and thin black lines are vegetation zones. From north to south the vegetation zones defined are: dry savanna (DS), mesic savanna (MS), transition zone (TZ), moist and dry semideciduous forest (SDF), rain forest (RF) and coastal savanna (CS).

We also disagree with Laris and Jacobs’s point as regards the apparent mistiming of early fire treatments in some West African experiments (Aubréville, 1953; Brookman‐Amissah *et al.,* 1980; Louppe *et al.,* 1995) especially as their Fig. 1 puts the line indicating the timing of early fire treatment in Central Ivory Coast in the wrong place: the correct date being the 2^nd^ half of December for Kokondekro (‘*2e quinzane de Décembre*’: Aubréville, 1953). Further, their Fig. 1 with fire incidence data taken solely from areas with a mean precipitation total >1.0 m yr^−1^ (Laris *et al.,* 2017) fails to take into account that across the West African savanna region the timing of the onset of the fire season varies in a systematic way with the expansion of the Harmattan from north to south. For example, in the Red Volta experimental region in Northern Ghana (which incidentally has a mean rainfall <1.0 m yr^−1^) November is the first major month of the fire season but for the more southerly forest–savanna transition zone area this is more typically later on in December and/or January. This is illustrated in our Fig. 1 where the seasonal trend of MODIS‐derived fire occurrences are shown along with the locations of the two fire trials in question: see also Owusu‐Afriyie (2008) and Janssen *et al*. (2018). As a rule of thumb, West African farmers start the fire season 2–4 wk after the last rainfall event, which is when the grass layer is combustible enough (a moisture content being necessary in the order of 30%) to set a self‐sustaining fire to it: see for example Finney *et al*. (2013) – and with the main peak logically following several weeks later. The early fire treatments in the experiments in Central Ivory Coast and Northern Ghana are thus not mistimed as Laris and Jacobs claim. Rather, they are actually very well timed indeed.

The Laris *et al*. (2017) paper does, however, make important observations on the importance of variations in soil resources and their influence on tree density in the Kokondekro trial. These wholly support our own analysis pointing out the importance of edaphic factors as important influences interacting with precipitation regime to modulate variations in tree cover extent and the position of the forest–savanna boundary in the tropics at local, continental and/or global scales (Lloyd *et al.,* 2015; Veenendaal *et al.,* 2015; Ametsitsi *et al.,* 2020; Gonçalves *et al.,* 2021), a concept that now seems to be becoming increasingly well appreciated (see, for example, Case & Staver, 2018).

Nevertheless, it is also clear that the results of Veenendaal *et al*. (2018) are not in agreement with several hypotheses recently presented regarding the effects of fires as influenced by time of burning and/or precipitation regime (Laris *et al.,* 2016, 2017). Perhaps more importantly, however, much of the ‘well‐established’ evidence that Laris and Jacobs present to support their arguments (Staver *et al.,* 2011a,b) should, in our view, be considered as little more than hypotheses arising from *in silico* interpretations of a remote sensing product with clear data fidelity issues: for extensive discussions of this issue see papers by Hanan *et al*. (2013), Staver & Hansen (2015), Veenendaal *et al*. (2015), Lloyd & Veenendaal (2016), Gerard *et al*. (2017), Wuyts *et al*. (2017), Gross *et al*. (2018), Kumar *et al*. (2019) and Adzhar *et al*. (2021). This contrasts with the Veenendaal *et al*. (2018) study which aimed to test the ASS theory for the forest–savanna transition using actual observational field data from all 11 quantifiable fire trials available to us. Even though geographically imbalanced, our analysis was not regionally limited and also included moist savanna sites on three continents. We analysed not only early and late fire treatments but also experiments with other fire timings to the extent that there was data available. Thus, although the large majority of data constituted early and late fire events there were also some with experimental fires in the middle part of the fire/dry season. Taken together these data were enough to give a significant trend of fire effects on vegetation cover increasing as the dry season progresses with no evidence that fire effects in the middle of the dry season should, as suggested in Laris *et al*. (2017), somehow have a higher impact than late in the dry season: see Fig. 2 of Veenendaal *et al*. (2018).

Moreover, in Laris *et al*. (2017) far reaching conclusions are drawn on the importance of midseason fire effects based only on incidental observations lacking any sort of statistically rigorous interpretation of the supposed treatment effects. By contrast, we presented a carefully constructed and defensible statistical tree cover modelling approach which included both seasonality and the frequency of fires as factors. We were also at pains to check model assumptions and, where possible, provided standard errors and/or confidence intervals for both model parameters and the ensuing model predictions.

We also note that none of the three papers cited by Laris and Jacobs as supposedly neglected by us, but purportedly providing supporting evidence for their notion that fire timing is much more important than frequency in determining woody cover effects of fire actually, shows what Laris and Jacobs claim to say they do. Specifically, the Devine *et al*. (2014) study (which uses data also included in our analysis) only examined effects of mid‐August fires of different frequencies, the Smit *et al*. (2016) study only examined the effects of annual fires in a management context, and with the Govender *et al*. (2006) study reporting only on fuel loads and fire intensities rather than longer terms effects of fire regime on woody vegetation structure for the South African Kruger plots that were included in our analysis in any case. In their same paragraph, Laris and Jacobs again show a lack of attention to detail in claiming that: ‘Of the studies used to examine the impacts of fire frequency, only the two sites from Australia had precipitation values above 1000 mm yr^–1^’, this obviously overlooking the fact that the South American IBGE fire trials as shown in Fig. 4 of Veenendaal *et al*. (2018) were obviously multiple frequency with the vegetation in question growing at a mean total precipitation of *c*. 1.45 m yr^−1^ (as clearly detailed in the associated Supporting Information section S1).

Finally as part of our response we would like to note that, as has also been misunderstood by others – see for example Staal & Flores (2015) and the subsequent response (Lloyd & Veenendaal, 2016) – we have never argued that fire does not have important impacts on vegetation structure (Veenendaal *et al.,* 2015, 2018; Lloyd & Veenendaal, 2016). The purpose of the paper by Veenendaal *et al*. (2018) was to simply quantify these fire effects in a statistically rigorous way and, based on that analysis, we concluded that it is unlikely that fire‐mediated feedbacks can lead to critical forest–savanna transitions and ASS.

Laris and Jacobs take issue with that conclusion but with the only evidence they cite as ‘demonstrating’ that ASS are already a documented phenomenon, namely Keeley & Rundel (2005) being a palaeostudy looking at simple vegetation–climate correlations. Moreover, the only tropical example in Keeley & Rundel (2005) is for East Africa at mean annual precipitations currently < 0.6 m yr^−1^ in an area where vegetation types are clearly influenced more by edaphic conditions than climate (White, 1983), and for which there is a current precipitation regime under which Laris and Jacobs themselves (amongst others) have argued that vegetation structure should be primarily determined by climate rather than ASS in any case. The corollary here seems to be that under the sort of disturbance regimes characteristic of the more mesic savannas (i.e. with mean annual precipitation ≥ 1.0 m yr^−1^) the occurrence of ASS should be more likely. Nevertheless, Van Langevelde *et al*. (2003) have suggested the opposite trend for more arid savannas with lower tree cover, and our own analysis suggests that for more mesic vegetation types fire can only maintain open savanna grasslands under deliberate human‐mediated, high‐frequency, late season fire regimes.
